# Cancer of the cervix uteri: 2025 update

**DOI:** 10.1002/ijgo.70277

**Published:** 2025-09-04

**Authors:** Neerja Bhatla, Daisuke Aoki, Daya Nand Sharma, Rengaswamy Sankaranarayanan

**Affiliations:** ^1^ Department of Obstetrics and Gynecology All India Institute of Medical Sciences New Delhi India; ^2^ Division of Preventive Medical Center Akasaka Sanno Medical Center Tokyo Japan; ^3^ International University of Health and Welfare Graduate School Tokyo Japan; ^4^ Department of Radiation Oncology All India Institute of Medical Sciences New Delhi India; ^5^ Division of Preventive Oncology Karkinos Healthcare, Kerala Operations Ernakulam India

**Keywords:** cancer, cervix, FIGO Cancer Report, HPV vaccination, immunotherapy, radiation, screening, staging, surgery

## Abstract

Since the publication of the 2021 FIGO Cancer Report, there has been further progress in the global effort to attain the WHO goal of cervical cancer elimination using a three‐pillar approach of vaccination, screening, and treatment. The HPV vaccination is now included in the national program of over 140 countries. Two‐dose schedules are being implemented in 80 countries and one‐dose schedules in 60 countries. Screening has seen major advances with the wider implementation of HPV testing, including the option of self‐sampling, portable screening and treatment devices, and the incorporation of artificial intelligence. Diagnostic accuracy is being enhanced with immunohistochemistry and receptor assays. Surgical treatment of invasive cancer is being revolutionized with the possibility of conservative surgery in very early stages, minimizing complications and adverse effects and offering feasibility of fertility sparing. New data on minimally invasive surgery are redefining the role of laparoscopy and robotic surgery in early stages with small tumor size. Sentinel lymph node evaluation is an emerging alternative to complete lymphadenectomy. Immunotherapy has opened up new possibilities for the management of recurrent and metastatic disease. This chapter discusses the management of cervical cancer based on the stage of disease, including attention to palliation and quality‐of‐life issues, with insights into the results from recent landmark trials.

## 1 INTRODUCTION

Globally, cervical cancer continues to be one of the most common cancers among women, being the fourth most common after breast, colorectal, and lung cancers. GLOBOCAN 2022 estimated that, worldwide, there were approximately 662 301 new cases of cervical cancer, with 348 874 deaths annually.^1^ The majority of new cases and deaths (approximately 85% and 90%, respectively) occur in low‐ and middle‐income countries (LMICs), where it is the third most common cancer among women.

## 2 ANATOMICAL CONSIDERATIONS

The cervix is the lowermost part of the uterus and is a cylindrical structure composed of stroma and epithelium. The ectocervix, which projects into the vagina, is lined with squamous epithelium. The endocervical canal, which extends from the internal os to the external os, is lined with columnar epithelium. Almost all cases of cervical carcinoma originate from the ecto‐ or endocervical mucosa in the transformation zone, the area of the cervix between the old and new squamocolumnar junction.

The accessibility of the cervix for visual inspection and sampling contributed to early understanding of the natural history of cervical cancer. The need for minimal or no anesthesia during treatments like freezing or cauterization enabled the development of simple outpatient screening and management techniques.

## 3 HPV EPIDEMIOLOGY

Cervical cancer is a rare, long‐term outcome of persistent infection of the lower genital tract with one of approximately 17 high‐risk HPV (hrHPV) types, which are recognized as the “necessary” cause of the disease.^2^, ^3^ Persistent HPV infection is defined by the repeated detection of the same type‐specific HPV DNA over a period of 6–12 months. More than 80% of women followed over time will acquire at least one hrHPV infection, showing its ubiquitous nature and ease of transmission. Approximately 90% of incident HPV infections are cleared within 2 years, with only approximately 10% persisting.^4^ It is these women who are at risk of developing cervical precancerous lesions. HPV types 16 and 18 account for 78% of cervical cancer cases, while types 31, 33, 45, 52, and 58 account for an additional 18%.^3^

It remains a matter of debate whether the virus is completely cleared when HPV tests return negative, or whether it persists in a latent state in basal cells, with the potential for reactivation in some cases. Recent publications have provided updated insights into the natural history of HPV infection and its clinical implications.^5^

## 4 PREVENTION AND EARLY DETECTION OF CERVICAL CANCER

Knowledge of HPV epidemiology and its role in cancer causation has resulted in the development of two major strategies for prevention and early detection, namely: (1) primary prevention by HPV vaccination; and (2) secondary prevention by screening and management of precancerous lesions.

WHO has launched a global initiative to eliminate cervical cancer as a public health problem, setting a pragmatic elimination threshold of four cases per 100 000 women. To achieve this goal by 2030, WHO has proposed a 90–70–90 triple‐pillar intervention strategy^6^:

- 90% of girls fully vaccinated with the HPV vaccine by the age of 15 years;
- 70% of women screened using a high‐performance test by the ages of 35 years and 45 years; and
- 90% of women with cervical lesions receiving appropriate treatment and care (treatment of precancers and management of invasive cancers).

Although high‐income countries (HICs) are already well advanced in implementing the above policy, the experience in LMICs is highly variable.

### 4.1 Primary prevention of cervical cancer with HPV vaccination

The estimated global, cross‐sectional HPV prevalence among healthy women aged over 30 years is approximately 11.7%, with the highest being in sub‐Saharan Africa at approximately 24% and country‐specific prevalence in the range of 2%–42% globally.^7^ Age‐specific cross‐sectional HPV prevalence peaks at 25% in women aged younger than 25 years, suggesting that the infection is predominantly transmitted through the sexual route after sexual debut. Thus, the prophylactic HPV vaccination should target women before the initiation of sexual activity, focusing on girls aged 9–14 years.

The HPV vaccination was launched in 2006. Three types of prophylactic HPV vaccines are currently available for use in girls and boys from the age of 9 years for the prevention of premalignant lesions and cancers affecting the cervix, vulva, vagina, and anus caused by hrHPV types: bivalent vaccines targeting HPV 16 and HPV 18; quadrivalent vaccines targeting HPV 6 and HPV 11 (for anogenital warts) in addition to HPV 16 and HPV 18; and the second generation nonavalent vaccine targeting five additional HPV types: 31, 33, 45, 52, and 58. All the vaccines are recombinant vaccines composed of virus‐like particles (VLPs) and are not infectious since they do not contain viral DNA. The development of two new WHO‐prequalified bivalent vaccines from China^8^ and a quadrivalent vaccine from India^9^ has added capacity to the global supply.

In December 2022, WHO further revised the previous dosage recommendations with an off‐label recommendation of one or two doses up to the age of 20 years.^10^ For the primary target group aged 9–14 years, as well as those aged 15 years and older, a one‐ or two‐dose schedule (at 0 and 6–12 months) is recommended. Those aged 21 years and older should receive two doses. Immunocompromised patients must receive three doses (at 0, 1–2, and 6 months).^10^ Dosage recommendations being followed globally are very variable. Currently, 80 countries are implementing two‐dose and >60 countries are implementing the single‐dose regimen in their national programs.^11^

The Journal of the National Cancer Institute (JNCI) monograph on the state of the science of single‐dose prophylactic HPV vaccination covers all aspects very comprehensively, including the evaluation of immunogenicity, efficacy, acceptability, cost‐efficacy, HIV populations, and modeling impact studies.^12^ Currently, the longest follow‐up study of a prospective cohort for an average period of 12 years has shown comparable efficacy with one dose compared to two and three doses.^13^ The number of participants in the one‐, two‐ (at 0 and 6 months), and three‐dose cohorts was 4949, 4980, and 4348, respectively, with 71%–82% in the different cohorts eligible to provide samples for genotyping. Vaccine efficacy against persistent HPV 16 and 18 infection was 92.0% (95% confidence interval [CI] 87.0–95.0) in the one‐dose arm, and comparable with that observed in two‐ (94.8%, 95% CI 90.0–97.3) and three‐dose arms (95.3%, 95% CI 90.9–97.5). No high‐grade precancer associated with HPV 16 and 18 was detected among vaccinated participants compared with eight precancers detected among the unvaccinated women.^13^

Safety of the HPV vaccines is reviewed on an ongoing basis, and the vaccines have been found to be safe with no adverse events more common in vaccinated individuals compared to the general population.^14^ Concerns regarding infertility and premature ovarian insufficiency have been expressed but are unfounded.^15^

At the population level, there is evidence for the effectiveness of HPV vaccination in terms of reduced prevalence of hrHPV types, anogenital warts, and high‐grade cervical abnormalities (CIN2+) caused by the vaccine types among young women, with some evidence of cross‐protection against non‐vaccine types as well.^16^ A systematic review and meta‐analysis involving 60 million individuals with follow‐up to 8 years after vaccination indicated that 5–8 years after vaccination, the following outcomes significantly declined: (1) prevalence of HPV 16 and 18 by 83% (relative risk [RR] 0.17, 95% CI 0.11–0.25) in girls aged 13–19 years and by 66% (RR 0.34, 95% CI 0.23–0.49) in women aged 20–24 years; (2) prevalence of HPV 31, 33, and 45 by 54% (RR 0.46, 95% CI 0.33–0.66) in girls aged 13–19 years; (3) anogenital warts by 67% (RR 0.33, 95% CI 0.24–0.46) in girls aged 15–19 years, by 54% (RR 0.46, 95% CI 0.36–0.60) in women aged 20–24 years, and by 31% (RR 0.69, 95% CI 0.53–0.89) in women aged 25–29 years. CIN2+ decreased significantly by 51% (RR 0.49, 95% CI 0.42–0.58) among screened girls aged 15–19 years and by 31% (RR 0.69, 95% CI 0.57–0.84) among women aged 20–24 years.^16^ Programs with multicohort vaccination and high vaccination coverage had a greater direct impact and herd effects.

The impact of HPV vaccination on significantly reducing the risk of invasive cervical cancer has also been shown recently in a Swedish follow‐up evaluation of 1 672 983 girls and women who were aged 10–30 years between 2006 and 2017.^17^ Cervical cancer was diagnosed in only 19 vaccinated women and in 538 unvaccinated women, with the highest reduction (88%) in girls vaccinated before the age of 17 and a reduction of 53% in those vaccinated at 17–30 years. Similar results are being reported from several countries.^18^, ^19^ In the UK, a review of the earliest national HPV vaccination program with the bivalent vaccine evaluated 13.7 million years of follow‐up of women aged 20–30 years. By June 30, 2019, there had been 448 fewer than expected cervical cancers and 17 235 fewer than expected cases of CIN3 in vaccinated cohorts in England, an estimated relative risk reduction of 87% and 97% in invasive cervical cancer and CIN3+, respectively, for girls vaccinated at 12–13 years. Corresponding figures in the age group 14–16 years were 62% and 75%, respectively, and in the age group 16–18 years were 34% and 39%, respectively. The authors concluded that the HPV immunization program has almost successfully eliminated cervical cancer in women born after September 1, 1995, in England.^19^

It has been estimated that worldwide HPV vaccination with high coverage could prevent approximately 8.7 million cases by 2094.^20^ There is no evidence of type replacement after vaccination.^21^

### 4.2 Secondary prevention of cervical cancer by early detection and treatment of precancerous lesions

Screening is an important strategy in the global elimination of cervical cancer. Although the HPV vaccination aims to prevent cervical neoplasia by preventing HPV infection, screening aims to detect prevalent cervical precancerous lesions, such as high‐grade CIN and adenocarcinoma in situ (AIS) early, and effectively treat them to prevent invasive cancer and decrease cervical cancer mortality rates. It will therefore remain a priority for cervical cancer prevention for several decades.

A review and synthetic analysis of cervical cancer screening programs and age‐specific coverage estimates for 202 countries and territories worldwide identified recommendations for cervical screening in 139/202 (69%) countries and territories.^22^ Cytology was the primary screening test in 109/139 (78%) countries, with 48/139 (35%) countries recommending primary HPV‐based screening. Visual inspection with acetic acid (VIA) was the most recommended test in resource‐limited settings. Estimated worldwide coverage in women aged 30–49 years in 2019 was 36% ever in lifetime. An estimated 1.6 billion (67%) of 2.3 billion women aged 20–70 years, including 662 million (64%) of 1.0 billion women aged 30–49 years, had never been screened for cervical cancer. The inequity was clear from the fact that 133 million (84%) of 158 million women aged 30–49 years living in HICs had been screened ever in lifetime, compared with 194 million (48%) of 404 million women in upper‐middle‐income countries, 34 million (9%) of 397 million women in LMICs, and only 8 million (11%) of 74 million in low‐income countries.

Cytology screening at regular intervals has resulted in a substantial decline in cervical cancer risk in HICs; however, it needs repeated rounds to compensate for poor sensitivity, which is resource‐intensive and not feasible in low‐resource settings. VIA, which involves the detection of acetowhite lesions on the cervix 1 min after the application of 3%–5% freshly prepared acetic acid, has been widely implemented in opportunistic settings in many low‐income countries in sub‐Saharan Africa and parts of Asia. Though feasible, it suffers from poor quality assurance and a high false positivity rate. HPV testing is unequivocally the best method of screening, with the highest sensitivity and the best negative predictive value, lower variability and better reproducibility compared with conventional or liquid‐based cytology. Sample collection by self‐sampling, essentially a high vaginal sample, has been shown to have good concordance with provider‐collected cervical sampling, making it feasible in remote areas.

The WHO guideline for screening and treatment of cervical pre‐cancer lesions for cervical cancer prevention has algorithms for several cervical screening strategies that have been used effectively in varied settings: cytology (conventional cervical smear and liquid‐based cytology [LBC]), HPV testing (DNA and mRNA); and VIA.^23^ These include algorithms for the general and HIV populations. The emphasis is on a single visit approach (SVA) to minimize loss to follow‐up, incorporating treatment at the screening visit whenever the lesion fulfills the stipulated criteria. SVA improves coverage, eliminates follow‐up visits, and improves cost‐efficiency in low‐resource settings.^24^, ^25^, ^26^ VIA is a suitable screening method for SVA, as is point‐of‐care HPV testing, especially with newer tests that incorporate partial genotyping to identify high‐risk types, such as HPV 16 and 18, in addition to other HPV types.

At present, there is a global effort to transition to HPV‐based primary screening. It was rolled out in Australia and the UK in 2017 and 2019, respectively. Many countries in Europe are also implementing HPV screening nationally or regionally, including the Netherlands, Sweden, Turkey, Finland, and Italy.^27^ Argentina, Chile, and Mexico are also implementing HPV‐based screening programs. Other countries in the process of transition include Thailand, Japan, and Sri Lanka. HPV screening has increased the colposcopy referral rates but also resulted in higher detection rates of CIN3+ lesions and cervical cancers.

Most guidelines recommend screening every 5 years for women after the age of 25–30 years, with variable endpoints of 64–74 years. The WHO call for elimination recommends only two lifetime screens by the ages of 35 and 45 years with a validated HPV test.^5^ In low‐resource settings, even a single lifetime HPV screening has been shown to be useful.^28^ In the context of vaccinated populations with declining HPV infections, primary HPV screening will be the solution, whose higher negative predictive value can allow extended screening intervals.^29^

Introducing a cervical cancer screening program in a country should be preceded by policy and managerial guidelines that clearly indicate the target age group, screening test and screening intervals, methods to reach target women, management of screen‐positive women (triaging and treating or SVA), treatment methods (cryotherapy, thermal ablation, large loop excision of the transformation zone [LLETZ]—also known as loop electrosurgical excision procedure [LEEP]) for CIN lesions, and criteria for the type of treatment for prevalent cervical cancers detected by screening.^23^, ^30^ Availability of adequate infrastructure and trained human resources is critical for initiating and sustaining a program. A program information system supported by a database and linkage with other information systems such as cancer registration, mortality registers, and health insurance databases is important for monitoring and evaluation. The screening strategy chosen must be feasible, simple, safe, accurate, acceptable, and easily accessible to the women at highest risk. Studies from Bangladesh and India observed that following the right approach to organize several components and meticulous attention to quality is crucial for the success of a screening program and not merely the choice of a good screening test.^31^ A judicious combination of HPV vaccination and screening has enormous potential to eliminate cervical cancer in the foreseeable future.

## 5 STAGING OF CERVICAL CANCER

Invasive cervical cancer spreads by direct extension into the parametrium, vagina, uterus, and adjacent organs, i.e. the bladder and rectum. It also spreads along the lymphatic channels to the regional lymph nodes, namely, the obturator, external iliac, internal iliac, and thence to the common iliac and para‐aortic nodes. Distant metastasis to the lungs, liver, and skeleton via the hematogenous route is a late phenomenon.

In 2018, the International Federation of Gynecology and Obstetrics (FIGO) Gynecologic Oncology Committee revised the staging to allow the option of clinical, radiological, or pathological findings, as available, to assign the stage. The revised staging is shown in Table 1.^32^ The main changes are:

- The horizontal dimension of a microinvasive lesion is no longer considered.
- Tumor size has been stratified further into three subgroups: IB1 ≤ 2 cm, IB2 > 2 – ≤4 cm, and IB3 > 4 cm.
- Lymph node positivity, which correlates with poorer oncologic outcomes assigns the case to Stage IIIC—pelvic nodes IIIC1 and para‐aortic nodes IIIC2. Micrometastases are included in Stage IIIC.

The revised FIGO staging is closely aligned with the latest TNM staging.^33^ The stage is allocated after all imaging and pathology reports are available. It is not to be altered later, for example at recurrence.

**TABLE 1:** FIGO staging of cancer of the cervix uteri (2018).

Stage			Description
I			The carcinoma is strictly confined to the cervix (extension to the uterine corpus should be disregarded)
	IA		Invasive carcinoma that can be diagnosed only by microscopy, with maximum depth of invasion ≤5 mm^a^
		IA1	Measured stromal invasion ≤3 mm in depth
		IA2	Measured stromal invasion >3 mm and ≤5 mm in depth
	IB		Invasive carcinoma with measured deepest invasion >5 mm (greater than Stage IA); lesion limited to the cervix uteri with size measured by maximum tumor diameter^b^
		IB1	Invasive carcinoma >5 mm depth of stromal invasion and ≤2 cm in greatest dimension
		IB2	Invasive carcinoma >2 cm and ≤4 cm in greatest dimension
		IB3	Invasive carcinoma >4 cm in greatest dimension
II			The carcinoma invades beyond the uterus, but has not extended onto the lower third of the vagina or to the pelvic wall
	IIA		Involvement limited to the upper two‐thirds of the vagina without parametrial involvement
		IIA1	Invasive carcinoma ≤4 cm in greatest dimension
		IIA2	Invasive carcinoma >4 cm in greatest dimension
	IIB		With parametrial involvement but not up to the pelvic wall
III			The carcinoma involves the lower third of the vagina and/or extends to the pelvic wall and/or causes hydronephrosis or non‐functioning kidney and/or involves pelvic and/or para‐aortic lymph nodes
	IIIA		The carcinoma involves the lower third of the vagina, with no extension to the pelvic wall
	IIIB		Extension to the pelvic wall and/or hydronephrosis or non‐functioning kidney (unless known to be due to another cause)
	IIIC		Involvement of pelvic and/or para‐aortic lymph nodes (including micrometastases),^c^ irrespective of tumor size and extent (with r and p notations)^d^
		IIIC1	Pelvic LNM only
		IIIC2	Para‐aortic LNM
IV			The carcinoma has extended beyond the true pelvis or has involved (biopsy proven) the mucosa of the bladder or rectum. A bullous edema, as such, does not permit a case to be allotted to Stage IV
	IVA		Spread of the growth to adjacent pelvic organs
	IVB		Spread to distant organs

Abbreviation: LNM, lymph node metastasis.

^a^ Imaging and pathology can be used, where available, to supplement clinical findings with respect to tumor size and extent, in all stages. Pathological findings supersede imaging and clinical findings.

^b^ The involvement of vascular/lymphatic spaces should not change the staging. The lateral extent of the lesion is no longer considered.

^c^ Isolated tumor cells do not change the stage but their presence should be recorded.

^d^ Adding notation of r (imaging) and p (pathology) to indicate the findings that are used to allocate the case to Stage IIIC. For example, if imaging indicates pelvic lymph node metastasis, the stage allocation would be Stage IIIC1r; if confirmed by pathological findings, it would be Stage IIIC1p. The type of imaging modality or pathology technique used should always be documented. When in doubt, the lower staging should be assigned.

## 6 HISTOPATHOLOGY

It is essential that all cancers must be confirmed by microscopic examination. Cases are classified as carcinomas of the cervix if the primary growth is in the cervix. The histopathologic types, as described in the WHO Classification of Female Genital Tumors,^34^ are as follows:

### 6.1 Squamous epithelial tumors

- Squamous cell carcinoma, HPV‐associated
- Squamous cell carcinoma, HPV‐independent
- Squamous cell carcinoma NOS

### 6.2 Glandular tumors

- Adenocarcinoma NOS
- Adenocarcinoma, HPV‐associated
- Adenocarcinoma, HPV‐independent, gastric type
- Adenocarcinoma, HPV‐independent, clear cell type
- Adenocarcinoma, HPV‐independent, mesonephric type
- Adenocarcinoma, HPV‐independent, NOS
- Endometrioid adenocarcinoma NOS

### 6.3 Other epithelial tumors

- Carcinosarcoma NOS
- Adenosquamous carcinoma
- Mucoepidermoid carcinoma
- Adenoid basal carcinoma
- Carcinoma, undifferentiated, NOS

### 6.4 Mixed epithelial and mesenchymal tumors

- Adenosarcoma

### 6.5 Germ cell tumors

- Endodermal sinus tumor
- Yolk sac tumor NOS
- Choriocarcinoma NOS

Neuroendocrine neoplasms (NENs) are classified separately in this classification. NENs are both aggressive and rare, with a high malignancy rate, high fatality rate, and poor prognosis. They are categorized as follows:

- Neuroendocrine tumor (NET)
- Neuroendocrine carcinoma (NEC) – small cell and large cell
- Carcinoma admixed with NEC

### 6.6 Use of immunohistochemistry for PD‐L1 expression and hormone receptor testing in cervical cancer diagnosis

Immunohistochemistry (IHC) now plays a critical role in diagnosis, prognostication, and treatment selection. Among various IHC markers, PD‐L1 expression and hormone receptor testing (estrogen receptor [ER]/progesterone receptor [PR]) are particularly important in specific histologic subtypes to guide immunotherapy and differential diagnosis.

#### PD‐L1 testing

PD‐L1 testing helps identify patients who may benefit from immune checkpoint inhibitors (ICIs), such as pembrolizumab. The 22C3 pharmDx assay is the IHC test for PD‐L1 expression in cervical cancer approved by the U.S. Food and Drug Administration.

Scoring system: PD‐L1 scoring is primarily calculated using the Combined Positive Score (CPS), which calculates the percentage of PD‐L1 positive tumor + immune cells (lymphocytes and macrophages) relative to the total number of viable tumor cells. A CPS of 1 or above is considered PD‐L1 positive, eligible for ICI therapy (e.g. pembrolizumab). A CPS of 10 or above suggests a higher likelihood of response to ICIs (Table 2).

**TABLE 2:** PD‐L1 expression in different histologic subtypes of cervical carcinoma.

Histologic subtype (proportion of cancers)	PD‐L1 expression	Therapeutic implication
SCC (75%–80%)	Frequently PD‐L1 positive (60%–80%)	Eligible for pembrolizumab in recurrent/metastatic cases
Adenocarcinoma (15%–20%)	Less frequently PD‐L1 positive (~20%–30%)	May still benefit from ICIs if CPS ≥1
Neuroendocrine carcinoma (5%)	Often PD‐L1 positive	Potential use of ICIs + chemotherapy
HPV‐independent gastric type adenocarcinoma	Rarely PD‐L1 positive	Limited response to immunotherapy

Abbreviations: CPS, complete positive score; ICI, immune checkpoint inhibitor; SCC, squamous cell carcinoma.

#### Hormone receptor testing in cervical cancer

Unlike endometrial cancer, most cervical cancers are not hormone driven. Thus, in cervical cancer, ER and PR testing is mostly useful for differentiating endocervical from endometrial adenocarcinoma and subtyping rare cervical adenocarcinomas.

In case the cancer cells express ER and PR, hormone receptor testing can play a crucial role in determining the potential responsiveness of a tumor to hormone therapy, thus influencing treatment planning and prognosis for the patient.

### 6.7 Distinction between HPV‐associated and HPV‐independent cervical cancer and its clinical relevance

Cervical cancer is now classified into HPV‐associated and HPV‐independent subtypes, which have distinct pathological, molecular, and clinical characteristics Table 3. Recognizing these differences is crucial for accurate diagnosis, prognostic assessment, and treatment planning.

**TABLE 3:** Differences between HPV‐associated vs. HPV‐independent cervical cancer.

Feature	HPV‐associated cancer	HPV‐independent cancer
Pathogenesis	Driven by HPV oncoproteins (E6, E7)	Genetic mutations (TP53, KRAS), hormonal influence
P16 IHC	Strong, diffuse positive	Negative or focal weak staining
HPV DNA/RNA testing	Positive	Negative
Histologic subtypes	Squamous cell carcinoma, usual‐type adenocarcinoma, adenosquamous carcinoma, small cell neuroendocrine carcinoma	Gastric‐type adenocarcinoma, clear cell carcinoma, mesonephric carcinoma, large cell neuroendocrine carcinoma
Response to treatment	Better response to chemoradiation	Poor response, requires multimodal therapy
Prognosis	Better prognosis	Worse prognosis, high recurrence

HPV infection rates differ between cervical squamous cell carcinoma and adenocarcinoma. Globally, 12.7% of squamous cell carcinoma and 15%–38% of cervical adenocarcinomas are HPV‐negative.^1^, ^2^

The common pathological types of HPV‐positive adenocarcinoma are intestinal, villoglandular, signet‐ring cell, and endometrioid adenocarcinoma, which originate from the cervical squamous columnar junction zone, accounting for nearly 90% of all cervical adenocarcinomas.^35^ The pathological types of HPV‐negative adenocarcinoma are gastric, clear cell, serous, and mesonephric adenocarcinomas. These types are quite rare and their occurrence might not be HPV‐related.

#### Clinical implications of this distinction

- HPV testing and p16 IHC are essential for accurate classification.
- HPV‐independent cancers often require alternative treatment strategies.
- HPV‐associated cervical cancer is largely preventable through vaccination and screening.

### 6.8 Risk stratification for lymph node involvement in cervical adenocarcinoma: The Elvio Silva Classification

The Elvio Silva Classification provides a histopathologic‐based system to assess the likelihood of lymphatic spread and recurrence, helping guide surgical and adjuvant treatment decisions. It stratifies HPV‐associated invasive endocervical adenocarcinoma into three patterns (A, B, and C) based on the presence or absence of destructive stromal invasion, degree of destructive stromal invasion (if present), presence or absence of lymphovascular space invasion (LVSI), and grade of cytologic atypia.

Pattern A has well‐demarcated glands without destructive stromal invasion and no LVSI. The risk of lymph node metastases (LNM) is less than 1%. Patients with pattern A tumors may be candidates for conservative or fertility‐sparing surgery, such as simple hysterectomy or conization with lymph node assessment omitted. No adjuvant therapy is required.

Pattern B has well demarcated glands with localized (limited, early) destructive stromal invasion with rare LVSI. The risk of LNM is approximately 5%. In patients with pattern B tumors, conservative surgery may still be an option, but lymph node evaluation (e.g. sentinel lymph node biopsy) may be considered. Adjuvant therapy is usually not required unless other high‐risk factors are present.

Pattern C demonstrates diffuse destructive stromal invasion with irregular glands and stromal response with frequent LVSI and perineural invasion. The risk of LNM is high (approximately 25%–35%). These patients require radical surgery (radical hysterectomy + lymphadenectomy) and often adjuvant therapy (chemoradiation). Sentinel lymph node biopsy (SLNB) or full pelvic lymph node dissection (PLND) is recommended.^36^

## 7 DIAGNOSIS AND EVALUATION OF CERVICAL CANCER

### 7.1 Microinvasive disease

Diagnosis of stages IA1 and IA2 is made on microscopic examination of a cone biopsy specimen, obtained by LLETZ or cold knife conization, which includes the entire lesion. It can also be made on a trachelectomy or hysterectomy specimen. The depth of invasion should not be greater than 3 mm or 5 mm, respectively, from the base of the epithelium. Note must be made of LVSI, which does not alter the stage, but may affect the treatment plan. The margins of the cone biopsy should be negative for disease. If the margins are positive for invasive cancer, the patient is allocated to stage IB1.^37^

### 7.2 Invasive disease

In the case of visible lesions, a punch biopsy will generally suffice for diagnosis; however, if it is not satisfactory, a small loop biopsy or cone may be required. Clinical assessment is the first step in the allocation of staging. FIGO 2018 staging permits the use of any of the imaging modalities according to available resources, i.e. ultrasound, computed tomography (CT), magnetic resonance imaging (MRI), or positron emission tomography (PET), to provide additional information on tumor size, nodal status, and local or systemic spread. MRI is the best method of radiologic assessment of primary tumors greater than 10 mm.^38^ However, ultrasound has also been shown to have good diagnostic accuracy in expert hands.^39^ The modality used in assigning staging should be noted for future evaluation. Imaging can identify additional prognostic factors that can guide the choice of the most appropriate treatment modality.

For the detection of nodal metastasis greater than 10 mm, PET‐CT is more accurate than CT and MRI, with false‐negative results in 4%–15% of cases.^40^ In areas with a high prevalence of tuberculosis and inflammation, especially HIV‐endemic areas, large lymph nodes are not necessarily metastatic. The clinician may make the decision on imaging or, when possible, can use fine‐needle aspiration or biopsy to exclude metastases. This is especially true in advanced stages, where surgical assessment of para‐aortic lymph nodes using minimally invasive surgery or laparotomy may be used to tailor treatment according to extent of disease. Surgical exclusion of para‐aortic lymph node involvement has been reported to correlate with prognosis better than radiographic exclusion alone.

A review of 22 articles that assessed the safety and impact of pretreatment para‐aortic lymph node surgical staging (PALNS) found that 18% (range 8%–42%) of patients with Stage IB–IVA cervical cancer (FIGO 2008) had para‐aortic LNM.^41^ The mean complication rate of PALNS was 9% (range 4%–24%), with lymphocyst formation being the most common. In another study, up to 35% of clinically assessed Stage IIB and 20% of Stage III tumors were reported to have positive para‐aortic nodes.^42^ In the FIGO 2018 staging, all these cases are assigned to Stage IIIC as lymph node involvement confers a worse prognosis.^43^ If only pelvic nodes are positive, it is Stage IIIC1; if para‐aortic nodes are also involved it is Stage IIIC2. A further notation must be added to indicate whether this allocation is based on only imaging assessment (r) or whether pathological confirmation is available (p). In due course, these data can be analyzed and reported accordingly.

FIGO no longer mandates any biochemical investigations or investigative procedures; however, in patients with frank invasive carcinoma, a chest radiograph and assessment of hydronephrosis (using renal ultrasound, CT, or MRI) should be carried out. The bladder and rectum are evaluated by cystoscopy and sigmoidoscopy, only if the patient is clinically symptomatic, but have largely been replaced by MRI evaluation. Cystoscopy may be considered in cases of a barrel‐shaped endocervical growth and where the growth has extended to the anterior vaginal wall. Suspected bladder or rectal involvement should be confirmed by biopsy and histologic evidence. Bullous edema alone does not warrant a case to be allocated to Stage IV.

## 8 MANAGEMENT OF CERVICAL CANCER

Management of cervical cancer primarily involves surgery or radiation therapy, with chemotherapy serving as a valuable adjunct. In recent years, immunotherapy has emerged as an option, particularly for recurrent disease.

### 8.1 Surgical management

Surgery is suitable for early stages, where cervical conization, simple hysterectomy, or radical hysterectomy may be selected according to the stage of disease. Table 2 shows the types of radical hysterectomy. In Stage IVA, selected cases may be suitable for pelvic exenteration.

#### Microinvasive cervical carcinoma: FIGO Stage IA

##### Stage IA1

The treatment is completed with cervical conization unless there is LVSI or tumor cells are present at the surgical margin. In women who have completed childbearing or in elderly women, an extrafascial hysterectomy may also be recommended.^44^ Any route can be chosen, i.e. abdominal, vaginal, or minimally invasive. When LVSI is evident, pelvic lymphadenectomy should be considered, along with an extrafascial hysterectomy.^45^ If fertility is desired, cervical conization with close follow‐up will be adequate.

##### Stage IA2

Since there is a small risk of LNM in these cases, pelvic lymphadenectomy is performed in addition to a type B radical hysterectomy.^46^, ^47^ In low‐risk cases (no LVSI, sentinel node negative), simple hysterectomy or trachelectomy, combined with either pelvic lymphadenectomy or sentinel lymph node assessment, may be adequate surgical treatment. When the patient desires fertility, she may be offered a choice of the following: (1) cervical conization with pelvic lymphadenectomy (open or minimally invasive surgery [MIS]); or (2) radical trachelectomy with pelvic lymphadenectomy by abdominal, vaginal, or MIS route.

##### Post‐treatment follow‐up

After fertility‐sparing surgery, follow‐up with 3–4 monthly Pap smears for 2 years, then 6‐monthly for the next 3 years is recommended. With normal follow‐up at 5 years, the patient can return to the routine screening schedule according to the national guidelines. Other tests, including imaging, are not recommended routinely and may be performed if required on a case‐by‐case basis.

#### Invasive cervical carcinoma: FIGO Stage IB1, IB2, and IIA1

Surgical treatment is the preferred modality for the treatment of Stage IB1, IB2, and IIA1 lesions (Table 4). The previous concept of type C radical hysterectomy with pelvic lymphadenectomy in these cases has witnessed considerable changes for early‐stage disease recently.

**TABLE 4:** Types of radical hysterectomy.

	Simple extrafascial hysterectomy	Modified radical hysterectomy	Radical hysterectomy
Piver and Rutledge classification	Type I	Type II	Type III
Querleu and Morrow classification	Type A	Type B	Type C
Indication	Stage IA1	Type IA1 with LVSI; IA2	Stage IB1 and IB2, selected Stage IIA
Uterus and cervix	Removed	Removed	Removed
Ovaries	Optional removal	Optional removal	Optional removal
Vaginal margin	None	1–2 cm	Upper one‐quarter to one‐third
Ureters	Not mobilized	Tunnel through broad ligament	Tunnel through broad ligament
Cardinal ligaments	Divided at uterine and cervical border	Divided where ureter transits broad ligaments	Divided at pelvic side wall
Uterosacral ligaments	Divided at cervical border	Partially removed	Divided near sacral origin
Urinary bladder	Mobilized to base of bladder	Mobilized to upper vagina	Mobilized to middle vagina
Rectum	Not mobilized	Mobilized below cervix	Mobilized below cervix
Surgical approach	Laparotomy or laparoscopy or robotic surgery	Laparotomy or laparoscopy or robotic surgery	Laparotomy or laparoscopy or robotic surgery

##### FIGO Stage IB1

FIGO Stage IB1 is considered low risk with the following criteria: cervical stromal invasion less than 50% and no suspicious lymph nodes on imaging. A modified radical hysterectomy may be considered in these cases. Pelvic lymphadenectomy should always be included on account of the high frequency of lymph node involvement.^46^, ^47^ A pelvic nerve‐sparing surgical procedure is now the standard recommendation in patients undergoing radical hysterectomy (type C1 hysterectomy), insofar as radical curability is maintained, as intrapelvic injuries to the autonomic nerves (i.e., hypogastric nerve, splanchnic nerve, and pelvic plexus) often lead to impairment of urination, defecation, and sexual function, and consequent deterioration of the postoperative quality of life.^48^, ^49^

The SHAPE trial, an open label non‐inferiority trial, compared oncologic outcomes and treatment‐related adverse events between radical hysterectomy and simple hysterectomy in low‐risk early‐stage cervical cancer, defined as a tumor with a diameter less than 2 cm, absence of LVSI, and stromal involvement. They found that simple hysterectomy was not inferior to radical hysterectomy with respect to the 3‐year incidence of pelvic recurrence (2.17% in the radical hysterectomy group and 2.52% in the simple hysterectomy group; 90% CI −1.62 to 2.32). The incidence of urinary retention in the simple hysterectomy group was lower than in the radical hysterectomy group within 4 weeks after surgery (0.6% vs. 11.0%; *P* < 0.001) and beyond 4 weeks (0.6% vs. 9.9%; *P* < 0.001).^50^

The ConCerv trial was a prospective, single‐arm, multicenter study conducted across 16 sites in nine countries between April 2010 and March 2019.^51^ It was designed to evaluate whether less radical surgeries could effectively manage early‐stage, low‐risk cervical cancer while minimizing morbidity. The selection criteria were as follows: FIGO 2009 stage IA2–IB1 cervical carcinoma; histology squamous cell carcinoma (any grade) or adenocarcinoma (grade 1 or 2); tumor size under 2 cm; no LVSI; depth of invasion below 10 mm; negative imaging for metastatic disease; and negative conization margins. Initial eligibility was determined through cervical conization, with one repeat conization permitted, if necessary. In addition, women who had an inadvertent simple hysterectomy with an unexpected postoperative diagnosis of cancer were eligible if they met the inclusion criteria and subsequently underwent pelvic lymph node dissection (Table 3).

Based on fertility preservation desires, participants were allocated to two groups: (1) the fertility preservation group: cervical conization followed by pelvic lymph node assessment, which included sentinel lymph node biopsy and/or full pelvic lymph node dissection; or (2) the non‐fertility preservation group: cervical conization followed by simple hysterectomy with pelvic lymph node assessment. A total of 100 evaluable patients were enrolled, with a median age of 38 years (range 23–67 years); 33% had Stage IA2, while 67% had Stage IB1. Surgical Interventions were as follows: conization followed by lymph node assessment (*n* = 44); conization followed by simple hysterectomy with lymph node assessment (*n* = 40); and inadvertent simple hysterectomy followed by lymph node dissection (*n* = 16). Positive lymph nodes were identified in 5 (5%) patients. Residual disease in the post‐conization hysterectomy specimen was noted in 1/40 patients, indicating an immediate failure rate of 2.5%. Over a median follow‐up of 36.3 months (range 0.0–68.3 months), three patients developed recurrent disease within 2 years of surgery, resulting in a cumulative incidence of 3.5% (95% CI 0.9–9.0).

The study findings suggest that conservative surgical approaches may be a feasible option for select patients with early‐stage, low‐risk cervical carcinoma, to achieve favorable oncologic outcomes while potentially reducing surgical morbidity and preserving fertility when desired.

Fertility‐sparing treatment strategies: In the management of young women with Stage IA2–IB1 tumors, less radical surgical procedures have been explored that balance oncologic safety with the desire for fertility preservation:

Conization: As discussed in the “Surgical management” section, removal of a cone‐shaped section of abnormal tissue from the cervix can be both diagnostic and therapeutic in Stage IA2.^52^

Radical trachelectomy: In this procedure, the cervix and the parametrium are removed followed by anastomosis of the uterus with the vaginal end, allowing for the possibility of future pregnancies. Trachelectomy can be carried out by open abdominal, vaginal, or minimally invasive routes.^53^, ^54^ When a vaginal approach is planned, pelvic lymph nodes are first removed laparoscopically and examined via frozen section to confirm node negativity before proceeding with radical vaginal trachelectomy. Alternatively, lymph node assessment may be performed using conventional pathology, with the radical trachelectomy scheduled as a second surgery approximately 1 week later.

Patient selection: Careful selection of candidates for fertility‐sparing surgery is crucial to ensure oncologic safety. Ideal candidates are typically younger women with early‐stage cervical cancer, tumors less than 2 cm in size, and no LVSI. A comprehensive preoperative evaluation, including imaging and possibly sentinel lymph node biopsy, is essential to assess the extent of disease and ensure suitability for conservative management.^55^

Recurrence rates: The systematic review by Nezhat et al.^56^ found a mean cancer recurrence rate of 3.2% and a cancer‐related death rate of 0.6% after a median follow‐up period of approximately 40 months, indicating that less radical procedures can be oncologically safe.

Reproductive outcomes: A systematic review of 65 studies with 3044 patients who underwent fertility‐sparing surgery reported a mean clinical pregnancy rate of 55.4%, with the highest rate observed after vaginal radical trachelectomy at 67.5%. The mean live‐birth rate across these studies was 67.9%.^56^

Although fertility‐sparing surgeries offer potential for future pregnancies, they are associated with risks, such as cervical stenosis, second‐trimester miscarriages, and preterm deliveries. These risks necessitate thorough preoperative counseling and close obstetric monitoring during pregnancy.

##### FIGO Stage IB2 and IIA1

In FIGO Stages IB2 and IIA1 cervical cancer, surgery or radiotherapy can be chosen as the primary treatment depending on other patient factors and local resources, as both have similar outcomes.^57^, ^58^ The advantages of surgical treatment are as follows: (1) it is feasible to determine the precise postoperative stage on the basis of histopathologic findings, thereby enabling individualization of postoperative treatment; (2) it is possible to treat cancers that are likely to be resistant to radiotherapy; and (3) it is possible to conserve ovarian function. Intraoperative transposition of the ovaries high into the paracolic gutters, away from the radiation field if required postoperatively, is also feasible. The preservation of ovarian and sexual function makes surgery the preferred treatment in younger women. Type C radical hysterectomy is the standard procedure for the treatment of cervical cancer, consisting of removal of the uterus, parametrium, upper vagina, and a part of the paracolpium, along with pelvic lymphadenectomy. As for the adjacent connective tissues, the anterior vesicouterine ligament (anterior and posterior leaf), lateral cardinal ligaments, and posterior uterosacral and rectovaginal ligaments are cut from the uterus at sufficient distances from their attachments to the uterus. Pelvic lymphadenectomy is an important component of this surgical procedure. The regional lymph node excision includes the parametrial nodes, obturator nodes, external, internal, and common iliac nodes. The route of surgery used was laparotomy or MIS, either laparoscopic or robotic. However, the Laparoscopic Approach to Cervical Cancer (LACC) trial, a randomized trial that compared overall survival (OS) after open surgery versus laparoscopy or robotic surgery in early‐stage cervical cancer, showed a decreased OS in the MIS group (3/312 vs. 19/319, hazard ratio [HR] 6.00, 95% CI 1.48–20.3; *P* = 0.004). Disease‐free survival events were also increased three‐fold in the MIS group (7/312 vs. 27/319, HR 3.74, 95% CI 1.63–8.58; *P* = 0.002). Rates of intraoperative complications did not differ by treatment received (11% in both). The most common sites of recurrence were as follows: in the open arm, the vaginal vault (3/7, 43%); in the MIS arm, pelvis (7/24, 29%), pelvis along with multiple sites in abdomen (7/24, 29%). The authors concluded that hysterectomy by a minimally invasive route was associated with higher rates of recurrence than the open approach in patients with early‐stage cervical cancer.^59^

Subsequent to the LACC trial, several multi‐institutional observational studies confirmed inferior survival outcomes with MIS. Melamed et al.^60^ conducted a nationwide observational study in the USA and demonstrated a 4‐year mortality of 9.1% among patients with cervical cancer treated with MIS and 5.3% among those who underwent open surgery (HR 1.65, 95% CI 1.22–2.22). Uppal et al.^61^ reported recurrence‐free survival outcomes for a cohort of 700 patients with open or MIS radical hysterectomy. After propensity matching, they found that the recurrence risk at 5 years was 6.1% with open surgery and 14.4% with MIS (HR 2.93, 95% CI 1.22–7.0). A European cohort study (SUCCOR) reviewed 1272 patients with FIGO Stage IB1 cancer and found that the risk of recurrence for MIS‐treated patients was twice as high as that with open surgery (HR 2.07, 95% CI 1.35–3.15).^62^ Paik et al.^63^ reviewed 738 women who underwent radical hysterectomy for FIGO Stage IB–IIA cervical cancer. They also demonstrated that MIS had inferior disease‐free survival compared with those who had open surgery (HR 2.74, 95% CI 1.3–5.7).^63^ All this evidence suggests that MIS for cervical cancer could result in an excess risk of recurrence or death compared with an open approach.

In the study by Uppal et al.,^61^ among tumors with a diameter less than 2 cm, 4.4% recurrences were noted in the open group versus 11.5% in the MIS group (HR 2.83, 95% CI 1.1–7.18; *P* = 0.019) and prior conization was associated with a lower risk of recurrence (4.9% vs. 16.2%; *P* = 0.001).

In response to the LACC trial, several ongoing clinical trials are evaluating safety measures in laparoscopic radical hysterectomy for cervical cancer to determine whether MIS can be performed without compromising oncologic outcomes.

1. Robotic Approach to Cervical Cancer (RACC) trial^64^: RACC is an ongoing prospective, multicenter, international, open‐label randomized controlled trial to assess whether robotic‐assisted radical hysterectomy can achieve non‐inferior oncologic outcomes compared to open radical hysterectomy while potentially offering benefits such as reduced perioperative morbidity and faster recovery in patients with early‐stage cervical cancer

Women aged over 18 years with FIGO Stage IB1, IB2, and IIA1 cervical cancer (squamous, adenocarcinoma, or adenosquamous) are eligible to participate. The primary endpoint is recurrence‐free survival at 5 years. The use of a uterine manipulator is not allowed, and closure of the vagina before colpotomy is recommended but not mandatory.

2 Robotic versus Open Radical Hysterectomy for Early‐Stage Cervical Cancer (ROCC) trial (GOG‐3043): ROCC is another ongoing multicenter, randomized, open‐label, non‐inferiority trial to compare robotic‐assisted radical hysterectomy to the open approach. Patients with FIGO Stage IA2, IB1, and IB2 cervical cancer without metastatic disease are eligible to participate. The primary endpoint is progression‐free survival (PFS) at 3 years.

The ROCC trial seeks to provide high‐level evidence on the safety and efficacy of robotic‐assisted radical hysterectomy compared to the traditional open approach.

3 Laparoscopic Approach to Uterine Cervical Neoplasia (LAUNCH) trial: LAUNCH is a series of clinical studies designed to evaluate the safety and efficacy of laparoscopic radical hysterectomy compared to abdominal radical hysterectomy in patients with cervical cancer. These are multicenter, prospective, randomized, open, blinded endpoint (PROBE) non‐inferiority controlled trials. Each trial focuses on different stages of cervical cancer, with the collective aim of providing comprehensive data on the role of laparoscopic surgery in managing cervical cancer across various stages and patient populations, potentially influencing future surgical guidelines and patient care strategies.

I LAUNCH 1 trial: Early‐stage cervical cancer (FIGO 2018 stages IA1 with LVSI and IA2).

II LAUNCH 2 trial: Patients with FIGO Stage IB1, IB2, and IIA1 cervical cancer.

III LAUNCH 3 trial: This is the third study of the LAUNCH trial series to evaluate whether there is a difference between laparoscopic and abdominal radical hysterectomy in cervical cancer (Stages IB3 and IIA2) patient survival under stringent operation standards and consistent surgical oncologic principles. The primary endpoint is 5‐year OS. Secondary endpoints are 5‐year PFS, recurrence, and quality of life measurements; oncologic safety and reproductive outcomes.

#### Specific surgical techniques evaluated to enhance the safety of MIS in cervical cancer

Some ongoing clinical trials evaluate the safety of certain strategies that aim to improve oncologic outcomes by mitigating the risks associated with MIS in radical hysterectomy for cervical cancer. The outcomes of these studies will be crucial in determining the future role of MIS in the surgical management of early‐stage cervical cancer.

- Avoidance of tumor manipulation: Intraoperative tumor manipulation has been implicated in the dissemination of cancer cells during MIS. To address this, alternative methods have been developed to handle the uterus without direct tumor contact. For instance, a technique involving the use of tagged uterine sutures instead of a uterine manipulator has been described. In this approach, the uterine fundus is tied with sutures, allowing for manipulation without direct contact with the tumor. In addition, tubal ligation is performed to prevent potential tumor spillage through the fallopian tubes. This method aims to reduce the risk of cancer cell dissemination during laparoscopic radical hysterectomy.^65^
- Prior cervical conization: Performing conization before radical hysterectomy has been suggested as a protective factor, potentially reducing tumor burden and the risk of intraoperative tumor spread. The SUCCOR study was a retrospective European observational study designed to evaluate oncologic outcomes in patients undergoing radical hysterectomy for early‐stage cervical cancer (FIGO 2009 stage IB1, tumor size ≤2 cm). The study primarily compared MIS versus open surgery and assessed the potential protective role of prior cervical conization before radical hysterectomy.^62^

There was no difference in oncologic outcome between the open and MIS approach in tumors less than 2 cm. Patients who underwent MIS using a uterine manipulator had 2.76 times higher risk of relapse (HR 2.76; 95% CI, 1.75–4.33; *P* < 0.001), whereas MIS with protective vaginal closure had similar rates of relapse as open surgery (HR 0.63; 95% CI, 0.15–2.59; *P* < 0.52). Prior conization before radical hysterectomy was associated with better oncologic outcomes: 65% lower risk of recurrence and 75% lower risk of death. Preoperative conization may serve as a protective factor, may reduce tumor burden and lower the risk of intraoperative tumor spillage during MIS procedures.

- Vaginal closure techniques: Another innovative technique to prevent tumor spillage during colpotomy in MIS is the “no‐look no‐touch” technique, which involves creating and closing a vaginal cuff before colpotomy, thereby concealing the tumor and minimizing exposure. This approach includes steps such as manipulation of the uterus without an intrauterine manipulator, and exposure of the paracervical tissues using a suspension technique. The goal is to reduce the risk of cancer cell dissemination during surgery.^66^

Laparoscopy in the open state: Another ongoing open label, two‐armed phase 3 randomized clinical trial by Zhao et al. is comparing laparoscopic radical hysterectomy in the open state with abdominal radical hysterectomy in patients with early‐stage cervical cancer. They aim to reduce the possibility of tumor proliferation and tumor cell dissemination while retaining minimally invasive by the following: (1) gasless laparoscopy surgery and abdominal wall suspension; (2) insert the port into the umbilical foramen to achieve abdominal pressure in the open state, and the smoke from the electrosurgical instrument can be smoothly discharged; (3) close the vaginal resection area before vaginal incision without the use of uterine manipulators. The primary endpoint is the rate of disease‐free survival at 4.5 years. Secondary aims include treatment‐related morbidity, costs and cost‐effectiveness, patterns of recurrence, quality of life, pelvic floor function, and OS.^67^

#### Role of sentinel lymph node mapping

The role of sentinel lymph node (SLN) mapping^68^, ^69^ in cervical cancer is finding increasing acceptance in early‐stage cervical cancer, i.e. FIGO Stages IA, IB1, and IB2. Dual labeling using blue dye and radiocolloid increases the accuracy of SLN detection.^70^ Indocyanine green dye with near infrared technique can be used in both the open and minimally invasive approaches. Pelvic lymphadenectomy needs to be considered if LVSI is present. In SENTICOL‐2, the first randomized trial of SLN resection alone versus SLN plus pelvic lymphadenectomy in early cervical cancer, no false‐negative case was observed in the SLN plus lymphadenectomy arm among 206 patients. Lymphatic morbidity was significantly lower in the SLN arm than in the SLN plus lymphadenectomy arm (31.4% vs. 51.5%; *P* = 0.0046), as was the rate of postoperative neurological symptoms (7.8% vs. 20.6%; *P* = 0.01, respectively).^71^ The 3‐year recurrence‐free survival was not significantly different between the two arms. Currently, the SENTICOL‐III study is ongoing, which has enrolled 989 patients, randomly assigned to SLN or SLN plus pelvic lymphadenectomy, with the aim of comparing disease‐free survival and health‐related quality of life outcomes between.^72^

#### FIGO Stages IB3 and IIA2

In Stages IB3 and IIA2, the tumors are larger and the likelihood of high‐risk factors such as positive lymph nodes, positive parametria, or positive surgical margins that increase the risk of recurrence and require adjuvant radiation after surgery are high. Risk factors that increase the risk of pelvic recurrence even when nodes are not involved include largest tumor diameter (>4 cm), LVSI, and invasion of the outer one‐third of the cervical stroma.^73^, ^74^ In such cases, adjuvant whole pelvic irradiation reduces the local failure rate and improves PFS compared with patients treated with surgery alone.^73^ However, combining both treatment modalities increases the risk of major morbidity for the patient.

The treatment modality must, therefore, be determined based on the availability of resources and tumor‐ and patient‐related factors. Concurrent platinum‐based chemoradiation (CCRT) is the preferred treatment option for Stages IB3 to IIA2 lesions. It has been demonstrated that the prognosis in terms of OS, PFS, and local and distant recurrences is more favorable with CCRT, rather than radical hysterectomy followed by radiotherapy as postoperative adjuvant therapy.^75^, ^76^

In areas where radiotherapy facilities are scarce, neoadjuvant chemotherapy (NACT) has been used with the following goals: (1) to downstage the tumor to improve the radical curability and safety of surgery; and (2) to inhibit micrometastasis and distant metastasis. There is no consensus as to whether it improves prognosis compared with the standard treatment. Two randomized trials, EORTC 55994^77^ and the study by Gupta et al.,^78^ had varied outcomes. The EORTC trial showed no difference in 5‐year OS between NACT and CCRT, though the NACT arm experienced more chemotherapy‐related toxicity. The study by Gupta et al.^78^ showed superior disease‐free survival in the CCRT arm. The extent of surgery after NACT remains the same, i.e., radical hysterectomy and pelvic lymphadenectomy. The greater difficulty is in determining the indications for adjuvant therapy, which are often kept the same as those after primary surgery.^73^, ^78^ However, it must be remembered that NACT may give a false sense of security by masking the pathologic findings and thus affecting evaluation of indications for adjuvant radiotherapy/CCRT. NACT surgery is best reserved for research settings or those areas where radiotherapy is unavailable. This is especially true in patients with very large tumors or adenocarcinoma, which have lower response rates.^79^

#### FIGO Stage IVA or recurrence

Rarely, patients with Stage IVA disease may have only central disease without involvement up to the pelvic sidewall or distant spread. Such cases, or in the case of such a recurrence, pelvic exenteration can be considered but usually has a poor prognosis.^80^, ^81^, ^82^

### 8.2 Radiation management

In LMICs, the majority of patients present with locally advanced disease, where surgery plays a limited role. Over the last two decades, development of sophisticated planning and delivery techniques, and introduction of computer technology and imaging have revolutionized the practice of radiotherapy, resulting in improved clinical outcomes and reduced toxicity.^83^, ^84^

Apart from its curative role, radiotherapy is used as adjuvant therapy for operated patients with risk factors to prevent locoregional recurrence, and as palliative therapy for alleviating distressing symptoms in patients with advanced incurable disease.

#### Radiation therapy for early‐stage disease (FIGO Stages IA, IB1, IB2, and IIA1)

Although surgery is preferred for early‐stage disease, in cases with contraindications for surgery or anesthesia, radiotherapy provides equally good results in terms of local control and survival.^57^ The treatment decision should be based on clinical, anatomic, and social factors. Patients with microinvasive disease have been treated by intracavitary radiation therapy (ICRT) alone with good results if surgery is contraindicated owing to medical problems. Selected patients with very small Stage IB1 disease (<1 cm) may also be treated with ICRT alone, particularly if there are relative contraindications to external beam radiation therapy (EBRT).^85^ A dose of 60–65 Gy equivalent is usually prescribed to Point A or high‐risk clinical target volume (HRCTV). A combination of EBRT and ICRT is also an option for such patients.

Definitive radiotherapy or CCRT is preferred in patients likely to require postoperative radiotherapy to avoid compounding treatment‐related morbidity. There is a single randomized trial comparing surgery and radiotherapy^57^ but none comparing surgery to CCRT, which is the current standard in patients treated by definitive radiotherapy. Landoni et al.^57^ randomized patients with Stage IB or IIA cervical cancer to surgery with or without postoperative radiotherapy (PORT) versus definitive radiotherapy alone. PORT was administered to 64% of patients in the surgery arm. The two treatment arms resulted in similar OS (83%) and disease‐free survival (74%); severe morbidity was higher in the surgery arm (28% vs. 12%), likely due to contributions from both treatment modalities. An update of the same trial with 20‐year follow‐up data has shown marginally better results with radiotherapy compared with surgery (77% vs. 72%; *P* = 0.280). Multivariate analysis confirmed that risk factors for survival are histopathologic type (*P* = 0.020), tumor diameter (*P* = 0.008), and lymph node status (*P* < 0.001).^86^

#### Adjuvant radiotherapy

After radical hysterectomy, PORT with or without chemotherapy is indicated for patients with adverse pathologic factors. According to various prognostic factors, patients may be categorized into high‐, intermediate‐, or low‐risk disease. High‐risk disease includes patients with either positive surgical margins or LNM or parametrial spread; such patients should be offered PORT with chemotherapy since the GOG 109 trial has shown OS advantage.^74^ Intermediate‐risk patients with any two of three factors (tumor size >4 cm, LVI, deep stromal invasion) require PORT and no chemotherapy should be offered to these patients. All other patients following radical hysterectomy are termed low‐risk disease patients and do not need any adjuvant therapy.

A tumor size of more than 4 cm is a well‐known risk factor. It was incorporated in the FIGO staging system (2008) as Stage IB2 and subsequently in the FIGO 2018 staging revision as Stage IB3. With the advent of fertility‐sparing surgery, it became clear that a tumor size more than 2 cm is a risk factor.^55^, ^87^, ^88^

Gemer et al.^89^ evaluated various clinical and pathologic risk factors that may reduce the rate of multimodality treatment of early cervical cancer. The authors observed that 89% of patients with tumors 2 cm or greater and LVSI received radiotherapy and 76% of patients with tumors 2 cm or greater and depth of invasion greater than 10 mm received radiotherapy. They suggested that in patients with early cervical cancer, evaluation of tumor size and LVSI before performing radical hysterectomy could help to tailor treatment and reduce the rate of employing both radical hysterectomy and chemoradiation.

PORT consists of whole pelvic EBRT to cover the tumor bed and draining lymph node areas. A dose of 45–50 Gy is usually prescribed. Intensity modulated radiation therapy (IMRT), an advanced and refined technique of irradiation, has been explored in the postoperative setting to reduce the toxicity.^90^, ^91^ A phase III trial^91^ revealed improved patient‐reported outcomes at week 5 with IMRT, with no difference after treatment completion. If available, the use of IMRT is encouraged to reduce the toxicity to normal tissues.

The role of vaginal brachytherapy boost after EBRT is not clear; however, it may be considered for patients with close or positive margins, large or deeply invasive tumors, parametrial or vaginal involvement, or extensive LVSI.^92^ Vaginal cuff brachytherapy is usually delivered by ovoids or cylinders to the upper one‐third of the residual vagina and should include two weekly fractions of high dose rate (HDR) brachytherapy of 6 Gy, each prescribed to 5 mm from the vaginal cylinder/ovoid surface.

#### Radiation therapy for FIGO Stages IB3 and IIA2

Although feasible, surgery as an initial treatment is not encouraged for patients with Stage IB3 and IIA2 disease, since 80% of them require PORT or CCRT. It is well known that the addition of adjuvant radiotherapy to surgery increases morbidity and thus compromises quality of life.^93^, ^94^ In addition, combined modality treatment will unnecessarily overburden the surgical and radiation facilities, which are already inadequate in low‐resource countries. Therefore, CCRT is the standard of care for Stage IB3 and IIA2 disease. CCRT includes external radiation and intracavitary brachytherapy.^92^

#### Radiation therapy for FIGO Stages IIB–IVA

CCRT is considered the standard treatment for patients with LACC, based on the results of large randomized trials that tested the addition of chemotherapy to pelvic radiation.^95^, ^96^, ^97^, ^98^, ^99^ These studies demonstrated that CCRT had a significant survival advantage of 10%–15% at 5 years after treatment compared with radiotherapy alone; it also reduced local and distant recurrence. A subsequent meta‐analysis showed a maximum benefit of chemoradiation of 6% in Stage IB2 (now termed IB3) to Stage IIB and only a 3% benefit in Stage IIIB patients.^100^

A once‐weekly infusion of cisplatin (40 mg/m^2^ weekly with appropriate hydration) for 5–6 cycles during external beam therapy is a commonly used concurrent chemotherapy regimen.^100^ For patients who are unable to receive platinum chemotherapy, 5‐fluorouracil based regimens are an acceptable alternative.^101^, ^102^, ^103^ Data on the toxicity associated with concurrent chemotherapy and extended field irradiation are limited.^103^

**FIGURE 1:**
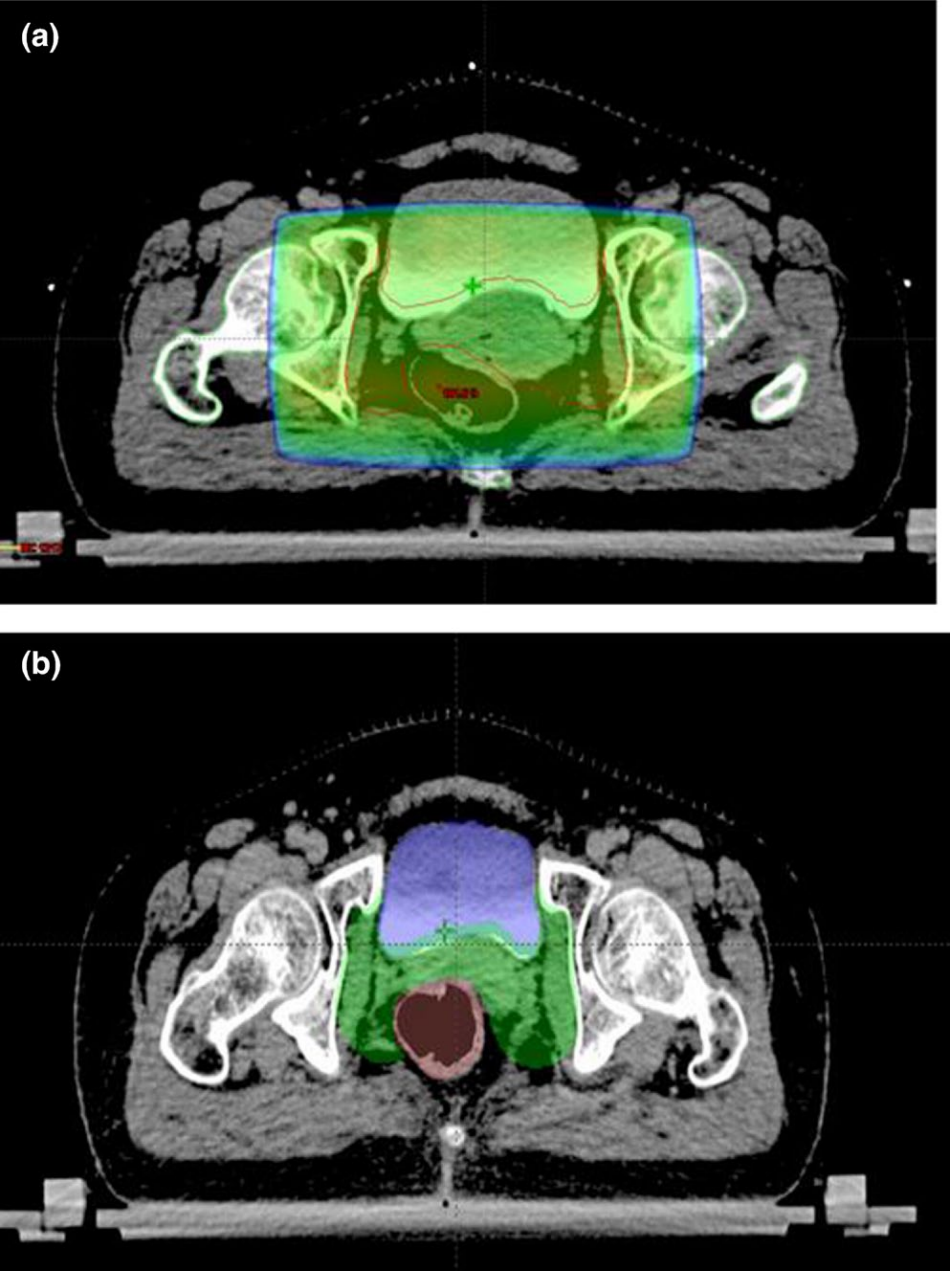
Computed tomography images showing radiotherapy planning using: (a) conventional four‐field box technique; and (b) IMRT planning. Normal tissues such as bladder and bowel are relatively spared in IMRT planning. *Note:* IMRT, intensity modulated radiation therapy.

The combination of EBRT and ICRT maximizes the likelihood of locoregional control while minimizing the risk of treatment complications. The primary goal of EBRT is to sterilize local disease and to shrink the tumor to facilitate subsequent ICRT. Standard EBRT should deliver a dose of 45–50 Gy to the whole pelvis by the 2‐ or 4‐field box technique (Table 5) encompassing the uterus, cervix, adnexal structures, parametria, and pelvic lymph nodes. Although EBRT is commonly delivered by a Cobalt‐60 teletherapy machine in several low‐resource countries, linear accelerators are now preferred as they provide higher‐energy beams resulting in more homogeneous dose delivery to deep tissues with relative sparing of superficial tissues. Recently, conformal radiotherapy techniques like three‐dimensional (3D)‐CRT and IMRT are increasingly being used with encouraging results in terms of reduced toxicity owing to relative sparing of normal tissues (Figure 1).

**TABLE 5:** Field design for the pelvic radiotherapy.

Field	Border	Landmark
AP‐PA fields	Superior	L4–5 vertebral interspace
	Inferior	2 cm below the obturator foramen or 3 cm inferior to distal disease, whichever is lower
	Lateral	1.5–2 cm lateral to the pelvic brim
Lateral fields	Superior	Same as AP‐PA field
	Inferior	Same as AP‐PA field
	Anterior	Anterior to the pubic symphysis
	Posterior	0.5 cm posterior to the anterior border of the S2/3 vertebral junction. May include the entire sacrum to cover the disease extent

Abbreviations: AP, anteroposterior; PA, posteroanterior.

The quality of radiotherapy is a crucial determinant of treatment outcomes in cervical cancer. Proper execution of radiotherapy techniques like IMRT and image‐guided adaptive brachytherapy (IGABT) significantly improves local control, survival, and toxicity profiles. The EMBRACE I study proved the feasibility and efficacy of MRI‐based IGABT in the treatment of LACC FIGO stage IB‐IVA.^104^ MRI‐based brachytherapy allows for personalized target delineation and organ‐at‐risk contouring, followed by dose optimization and tailored multiparametric dose prescription. Results have shown high rates of local control compared to historical series that utilized point A‐based brachytherapy with 5‐year local control of 92%. This enhanced local control is likely attributed to advancements in target contouring, implant techniques, and 3D treatment planning. EMBRACE 1 also investigated treatment‐related late morbidity and patient‐reported outcomes. Their findings indicate that severe morbidity (grade ≥3) related to gastrointestinal, genitourinary, and vaginal toxicity is comparatively lower than retrospective studies. The 5‐year incidence of grade 3–5 morbidity is as follows: 6.8% for genitourinary events, 8.5% for gastrointestinal events, 5.7% for vaginal events, and 3.2% for fistulae.^105^

Standard ICRT is usually performed using a tandem and two ovoids, or a tandem and ring. Any of the dose‐rate systems, namely low‐dose‐rate (LDR), high‐dose‐rate (HDR), or pulsed‐dose‐rate (PDR), may be practiced as all three yield comparable survival rates.^105^ The dose is usually prescribed to point A or to high‐risk clinical target volume (HRCTV) if image‐based planning is used.

With an LDR system, a dose of 30–40 Gy is prescribed in one or two sessions. With HDR, various dose fraction schedules are used, employing a dose of 5.5–8 Gy by 3–5 weekly fractions. Owing to resource constraints and long traveling distances in low‐resource countries, delivering three instead of five fractions is often more realistic and allows for treatment of a higher number of patients. In the COVID era, hypofractionation (increase dose per day and reduce the number of fractions) was helpful to reduce the number of hospital visits. The total combined dose with EBRT and ICRT should be in the range of 80–90 Gy. Although PDR is rarely used, the overall treatment time and dose in PDR remains almost the same as in LDR except that the treatment is given in multiple hourly pulses each lasting for a few minutes.

**FIGURE 2:**
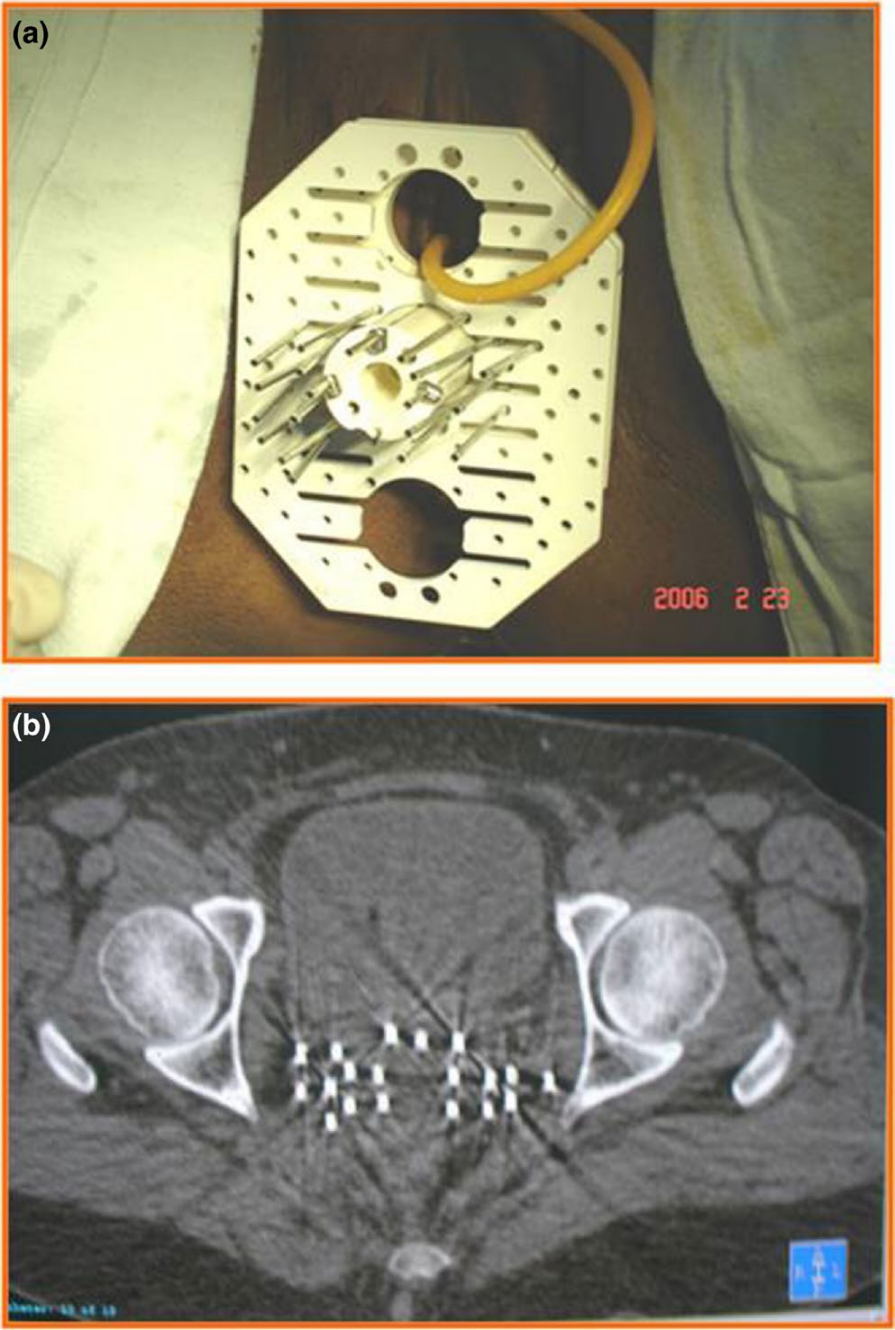
Interstitial brachytherapy implant: (a) clinical image of a patient showing the perineal template and the steel needles; and (b) computed tomography image showing the brachytherapy needles inserted into the pelvis.

If ICRT is not feasible, either due to distorted anatomy or inadequate dosimetry, then interstitial brachytherapy should be considered. Interstitial brachytherapy consists of inserting multiple needles/catheters into the primary tumor and parametria (Figure 2) through the perineum with the help of a template. Due to the risk of trauma to normal structures like the bowel and bladder, the use of ultrasound imaging (especially transrectal) is suggested during the implant procedure.^106^

Completion of the radiotherapy protocol within the stipulated time is an important goal as it has a direct correlation with outcome. In retrospective analyses, patients whose radiotherapy treatment times exceeded 9–10 weeks had significantly higher rates of pelvic failure when compared with women whose treatment was completed in less than 6–7 weeks.^107^, ^108^ The current recommendation is to complete the entire protocol of EBRT and brachytherapy within 8 weeks.

The OUTBACK study, an international randomized controlled trial, explored the role of additional adjuvant carboplatin and paclitaxel chemotherapy given after standard cisplatin‐based chemoradiotherapy (CRT) for unselected LACC. The study demonstrated increased short‐term toxicity without improvement in OS.^109^ In contrast, the GCIG INTERLACE trial explored the potential of short‐course induction chemotherapy, followed within 7 days by chemoradiation, to enhance treatment outcomes in LACC.^110^ By strategically delivering chemotherapy before CRT, this approach seeks to optimize tumor response by reducing tumor burden and eradicating micrometastatic disease, thereby improving long‐term disease control and patient survival. Patients with para‐aortic lymph node involvement and lower vaginal involvement were excluded. After a median follow‐up of 67 months, 5‐year PFS rates were 72% in the induction chemotherapy with CRT group and 64% in the CRT alone group (HR 0.65, 95% CI 0.46–0.91; *P* = 0.013). The 5‐year OS rates were 80% in the induction chemotherapy with CRT group and 72% in the CRT alone group (HR 0.60, 95% CI 0.40–0.91; *P* = 0.015). Distant‐only metastases were fewer in the induction chemotherapy arm (7% vs. 12%). However, it is to be noted that the INTERLACE trial started before the advent of modern precision RT. The survival rates in the standard CCRT arm of this trial are inferior to some recent reports. Moreover, the quality‐of‐life scores did not show any clinically relevant difference between the two treatment arms in the INTERLACE trial, indicating that the addition of induction chemotherapy before chemoradiation did not adversely impact patients' overall well‐being and functional outcomes. In low‐resource settings where access to high‐quality radiotherapy is constrained with a prolonged waiting time, the INTERLACE induction chemotherapy regimen can be carefully scheduled to align with confirmed radiotherapy dates, ensuring a seamless transition between treatments and preventing any unnecessary delays in care. This provides an effective alternative to enhance treatment outcomes.

#### Immunotherapy in locally advanced cervical cancer

Recent clinical trials have explored the integration of ICIs with CRT in the management of LACC.

KEYNOTE‐A18 (NCT 04221945) was a phase III, randomized, double‐blind, placebo‐controlled trial that recruited 1060 patients with newly diagnosed, high‐risk LACC (FIGO 2014 Stages IB2–IIB with positive lymph nodes and Stages III–IVA). Patients received either pembrolizumab (200 mg every 3 weeks for five cycles) or placebo, in combination with standard CRT, followed by maintenance pembrolizumab (400 mg every 6 weeks for 15 cycles) or placebo. The primary endpoint was PFS.^111^

At a median follow‐up of 29.9 months, the 24‐month PFS was 67.8% in the pembrolizumab plus CRT group versus 57.3% in the CRT alone group (HR 0.70, 95% CI 0.55–0.89; *P* = 0.002). The 36‐month OS rate was 82.6% with pembrolizumab plus CRT versus 74.8% with CRT alone, indicating a 33% reduction in the risk of death (HR, 0.67; 95% CI, 0.50–0.90; *P* = 0.0040). The authors concluded that the addition of pembrolizumab to CRT significantly improved PFS and OS in patients with high‐risk LACC, establishing this combination as a new standard of care.

The NICOL trial (NCT03298893) is a phase I, prospective, multicenter, dose‐confirmation trial that recruited 16 patients with Stage IB3‐IVA LACC. Patients received nivolumab (240 mg every 2 weeks) concurrently with CRT, followed by maintenance nivolumab. The primary endpoints were safety and tolerability.

With a median follow‐up of 16.6 months, three patients experienced disease progression at 3, 4, and 5 months, respectively. The NICOL trial established the safety and tolerability of combining nivolumab with CRT in patients with LACC, warranting further studies to assess efficacy.^112^

A recent meta‐analysis of phase 3 clinical trials (KEYNOTE‐826, CALLA, BEATcc, and ENGOT‐cx11/GOG‐3047/KEYNOTE‐A18) involving 2857 patients analyzed the trials for the efficacy and safety of adding ICIs to first‐line standard therapy (ST) for patients with recurrent or advanced cervical cancer. The analysis concluded that combining ICIs with ST not only offers superior efficacy over ST alone but also maintains a comparable toxicity profile, suggesting an effective and relatively safe treatment approach for managing this disease.^113^

Subgroup analyses within the meta‐analysis revealed that the combination of ICIs with ST significantly improved PFS, OS, and objective response rate (ORR) in patients with PD‐L1‐positive tumors. These findings underscore the importance of PD‐L1 expression as a critical biomarker for identifying patients who are likely to benefit the most from this treatment approach.^113^

#### FIGO Stage IVB/distant metastases

Presentation with distant metastatic disease is rare, reported in approximately 2% of cases. A management plan should consider that the median duration of survival with distant metastatic disease is approximately 7 months.

Concurrent chemoradiation may have a better response than systemic chemotherapy with overall and disease‐free survivals of 69% and 57%, respectively, reported in patients with positive para‐aortic and supraclavicular lymph nodes.^114^ Currently, there is no role for prophylactic extended field radiotherapy (EFRT) in LACC.^103^ When para‐aortic nodes are involved, EFRT with concurrent chemotherapy should be used. IMRT may be used in such patients to reduce the toxicity.

Despite limited response rates, cisplatin has been the standard chemotherapy used in the setting of distant metastatic disease.^115^ Recent evidence supports the use of platinum doublets over cisplatin alone, although with very modest benefits in response rates. Cisplatin may be combined with taxanes, topotecan, 5‐fluorouracil, gemcitabine, or vinorelbine.^116^ A carboplatin‐paclitaxel combination has also been successful in these cases. The possibility for immunotherapy in the management is supported by survival benefits in patients with advanced, metastatic, and recurrent cervical cancer, which has been recently reviewed.^117^

#### Radiation therapy after inadvertent incomplete surgery

Invasive cervical cancer may be found during pathologic evaluation of the specimen from a simple hysterectomy for an apparently benign condition. Inadvertent simple hysterectomy is considered inadequate surgery for invasive cervical carcinoma and subsequent therapy is required for all such cases. In such a situation, the extent of the disease should be assessed by a PET/CT scan if available, or a pelvic and abdominal CT or MRI scan, and chest imaging. The subsequent treatment plan is formulated based on the histologic and radiologic findings.

Although PORT for patients after inadvertent simple hysterectomy has been shown to be beneficial, the outcome for such patients even after PORT remains very poor, with a 5‐year recurrence‐free survival of 49%; therefore, CCRT is generally added.^118^, ^119^ In a study from India, Sharma et al.^118^ reported the results of 83 patients treated with PORT after either inadvertent simple hysterectomy (*n* = 33) or radical hysterectomy (*n* = 50). The 5‐year recurrence‐free survival was inferior in patients who underwent PORT after inadvertent simple hysterectomy (49% vs. 72%, respectively; *P* = 0.04).^118^ PORT, therefore, does not compensate for lack of adequate surgery.

In centers where the expertise is available, some of these patients may be found suitable for repeat laparotomy with parametrectomy and pelvic lymphadenectomy. The procedure is challenging due to previous scarring, adhesions, and distortion of anatomy, but does have the potential for curative surgery as well as to allow assessment of the need for adjuvant CCRT.^120^

### 8.3 Post‐treatment follow‐up

In a systematic review of 17 retrospective studies that followed up women treated for cervical cancer, the median time to recurrence was in the range of 7–36 months after primary treatment.^121^ Therefore, close clinical follow‐up in the first 2–3 years after treatment is important. Routine follow‐up visits are recommended every 3–4 months for the first 2–3 years, then 6‐monthly until 5 years, and then annually for life. At each visit, history taking and clinical examination are carried out to detect treatment complications and psychosexual morbidity, as well as assess for recurrent disease.

Routine imaging is not indicated. Special circumstances, such as involved high pelvic lymph nodes, may justify interval imaging of the abdomen to assess for potentially curable progression of disease. In the systematic review, asymptomatic recurrent disease was detected using physical exam (29%–71%), chest X‐ray (20%–47%), CT scan (0%–34%), and vaginal vault cytology (0%–17%). Frequent vaginal vault cytology does not significantly improve the detection of early disease recurrence.

Women aged under 50 years who have lost ovarian function should be considered for menopausal hormone therapy (MHT). As women age, the routine examination should also include other age‐indicated well‐woman checks to ensure quality of life, including assessment of thyroid and renal status.

### 8.4 Recurrent disease

Recurrences may occur locally in the pelvis, para‐aortic lymph nodes, distant metastases, or a combination thereof. The risk of both pelvic and distant failure increases in proportion to tumor volume.^122^ Most recurrences are seen within 3 years and prognosis is poor as most patients die from progressive disease, with uremia being the most common terminal event. The treatment plan depends on the patient's performance status, site, and extent of recurrence and/or metastases, and prior treatment received. Confirmation of recurrence with a pathologic specimen obtained by biopsy is essential before proceeding with therapy.

#### Management of local recurrence

The pelvis is the most common site of recurrence. Good prognostic factors are the presence of an isolated central pelvic recurrence with no involvement of the pelvic sidewall, a long disease‐free interval from previous therapy, and largest diameter of the recurrent tumor less than 3 cm.^123^

When the pelvic relapse follows primary surgery, it may be treated by either radical chemoradiation or pelvic exenteration. Radical irradiation with or without concurrent chemotherapy may result in 5‐year disease‐free survival rates in the range of 45%–74% with isolated pelvic failure after primary surgery.^124^, ^125^ The extent of recurrent disease and involvement of pelvic lymph nodes are prognostic factors for survival. Concurrent chemotherapy with either cisplatin and/or 5‐fluorouracil may improve outcome.

Pelvic exenteration may be feasible in some patients in whom there is no evidence of intraperitoneal or extrapelvic spread, and there is a clear tumor‐free space between the recurrent disease and the pelvic sidewall.^80^, ^81^, ^82^ Owing to its high morbidity, it is reserved for those with expected curative potential and requires careful patient selection regarding the associated physical and psychological demands. A PET/CT scan is the most sensitive non‐invasive test to determine any sites of distant disease and should be performed before exenteration, if possible.^126^, ^127^, ^128^ Patient assessment and counseling regarding the implications and ability to manage stoma and ostomy sites must also be addressed before surgery.^129^ The OS is 10% but careful selection of patients has been reported to yield a 5‐year survival with pelvic exenteration in the order of 30%–60%, and an operative mortality of less than 10%.^80^, ^81^, ^82^, ^130^

#### Para‐aortic nodal recurrence

The second most common site of recurrence is in the para‐aortic lymph nodes. Where there is isolated para‐aortic nodal recurrence, curative‐intent radiation therapy or chemoradiation can achieve long‐term survival in approximately 30% of cases.^131^, ^132^

#### Extensive local disease or distant metastases

If there is extensive local disease or distant metastatic disease, the patient is assigned to palliative therapy, with best supportive care. However, patients with an Eastern Cooperative Oncology Group performance status of 0–2 and only limited metastatic disease may be considered for palliative systemic chemotherapy. Where feasible, these patients could be offered participation in clinical trials, especially when the interval to relapse is less than 12 months.

A trial of platinum doublet chemotherapy along with bevacizumab is justified as depicted in the GOG 240 trial, which studied the efficacy of antiangiogenic therapy with bevacizumab—a humanized anti‐VEGF monoclonal antibody. When incorporated in the treatment of recurrent and metastatic cervical cancer, it showed increased OS (17.0 vs. 13.3 months, HR for death 0.71, 98% CI 0.54–0.95; *P* = 0.004).^133^ The treatment is expensive, and patients and their families need to be counseled on the limited benefits in terms of response rate and PFS. Adverse effects include increased incidence of hypertension, thromboembolic events, and gastrointestinal fistula.

In KEYNOTE‐826, patients with persistent, recurrent, or metastatic cervical cancer were randomized to receive pembrolizumab versus placebo in addition to platinum‐based chemotherapy and, per investigator discretion, bevacizumab. PFS and OS were significantly longer with pembrolizumab (PFS 10.4 months and 8.1 months, respectively, HR 0.58, 95% CI 0.44–0.77; *P* < 0.001; OS at 24 months 53% vs. 41.7%). The most common grade 3–5 adverse events were anemia (30.3% in the pembrolizumab group and 26.9% in the placebo group) and neutropenia (12.4% and 9.7%, respectively).^134^

In EMPOWER Cervical‐1, survival was significantly longer with cemiplimab than with single‐agent chemotherapy among patients with recurrent cervical cancer after first‐line platinum‐containing chemotherapy, in both squamous and adenocarcinoma (12.0 months vs. 8.5 months, HR for death 0.69; 95% CI 0.56–0.84; two‐sided *P* < 0.001).^135^

Although the patient populations differed in these trials, and approval status and availability may vary by country, it is important to note that the efficacy of ICIs in cervical cancer has been demonstrated. Second‐ or third‐line treatment with tisotumab vedotin resulted in significantly longer median OS than chemotherapy.^136^

#### Comprehensive palliative care

Symptom control is the essence of palliative care and plays a major role in maintaining dignity and quality of life. Common symptoms and signs of advanced cervical cancer include pain, ureteric obstruction causing renal failure, hemorrhage, malodorous vaginal discharge, lymphedema, and fistula. Patients require support from the corresponding clinical services as well as psychosocial care and support for their families and caregivers. Typically, a tiered approach to pain is practiced. Access to oral morphine is improving within LMICs and is an important aspect of palliative care. The availability of homecare teams in many regions and involvement of non‐governmental organizations in this effort can help minimize the need to transport the patient to hospital and save costs. In terminal cases, some patients may also require the services of a hospice facility.

#### Palliative radiotherapy

Short‐course radiotherapy is very effective in palliation of distressing symptoms. Although there is no standard dose fraction schedule, a dose of 20 Gy in five fractions over 1 week or 30 Gy in 10 fractions over 2 weeks is commonly practiced. In patients with severe vaginal bleeding, a short course of EBRT may be tried and, if it fails, ICRT can be highly effective in controlling the intractable bleeding.^137^ Control of bleeding is usually achieved after 12–48 h of radiotherapy.

In patients with pain arising from enlarged para‐aortic or supraclavicular nodes, skeletal metastases,^138^ and symptoms associated with cerebral metastases, palliative radiotherapy should be given via larger fractions over shorter periods of time. Commonly used schedules include large single fractions, 20 Gy in five fractions, and 30 Gy in 10 fractions.

## 9 SPECIAL SITUATIONS

### 9.1 Cervical cancer during pregnancy

Adequate management of these patients requires a multidisciplinary team. The plan must be discussed with the patient, and ideally her partner, to respect their wishes.

Broadly, the management of cervical cancer in pregnancy follows the same principles as in non‐pregnant patients. Before 16–20 weeks of gestation, patients are treated without delay. The mode of therapy can be either surgery or chemoradiation depending on the stage of the disease. Radiation often results in spontaneous abortion of the conceptus. From the late second trimester onward, surgery and chemotherapy can be used in selected cases while preserving the pregnancy.^139^, ^140^ When the diagnosis is made after 20 weeks, delaying definitive treatment is a valid option for Stages IA2, IB1, and 1B2 and has not been shown to have any negative impact on the prognosis compared with non‐pregnant patients.^141^ Timing of delivery requires a balance between maternal and fetal health interests. When delivered at a tertiary center with appropriate neonatal care, delivery by classical cesarean section and radical hysterectomy at the same time is undertaken no later than 34 weeks of gestation.

For more advanced disease, the impact of treatment delay on survival is not known. NACT may be administered to prevent disease progression in women with LACC when a treatment delay is planned.^141^, ^142^

## AUTHOR CONTRIBUTIONS

All authors contributed to the manuscript at all stages including design, planning, data abstraction, and manuscript writing.

## CONFLICT OF INTEREST STATEMENT

NB has received research funding through her institute from MSD, GlaxoSmithKline, Serum Institute of India Pvt. Limited, and Digene/Qiagen Inc. Outside of the submitted work, DA has received honoraria from AstraZeneca, Chugai Pharmaceutical, Eisai, Taiho Pharmaceutical, Coviden Japan, Johnson & Johnson, Genmab, and Takeda Pharmaceutical and consulting fees from Takeda Pharmaceutical, AstraZeneca, MSD, and Chugai Pharmaceutical. RS advises on cancer prevention and early detection for Karkinos Healthcare Private Limited, a managed healthcare company in India. DNS has no conflicts of interest to declare.

## Data Availability

Data sharing is not applicable to this article as no new data were created or analyzed in this study.
